# Prediction of Preadolescent Overweight and Poor Cardiometabolic Outcome in Children up to 6 Years of Age: Research Protocol

**DOI:** 10.2196/resprot.5158

**Published:** 2016-06-23

**Authors:** Marlou LA de Kroon, Alet Wijga, Yvonne Vergouwe, Martijn W Heijmans, Vincent WV Jaddoe, Jos WR Twisk, Hein Raat

**Affiliations:** ^1^ Erasmus University Medical Center Department of Public Health Rotterdam Netherlands; ^2^ National Institute of Public Health and the Environment Prevention and Health Services Bilthoven Netherlands; ^3^ The EMGO Institute for Health and Care Research VU University Medical Center Amsterdam Netherlands; ^4^ Erasmus University Medical Center The Generation R Study Group Rotterdam Netherlands

**Keywords:** overweight, (pre-)hypertension, High-Density Lipoproteins, forecasting, dynamic risk estimation, Child Health Services, pediatrics, prevention

## Abstract

**Background:**

Dynamic risk estimations may enable targeting primary prevention of overweight and overweight-related adverse cardiometabolic outcome in later life, potentially serving as a valuable addition to universal primary prevention. This approach seems particularly promising in young children, as body mass index (BMI) changes at a young age are highly predictive of these outcomes, and parental lifestyle interventions at a young age are associated with improved long-term outcome.

**Objective:**

This paper describes the design of our study, which aims to develop digitized tools that can be implemented in the Dutch Child Health Care (CHC) system or by pediatricians for children up to 6 years of age. These tools will enable (1) dynamically predicting the development of overweight, hypertension or prehypertension, low high-density lipoprotein cholesterol (HDL-C) values, and high total cholesterol to HDL-C ratio by early adolescence and (2) identifying children who are likely to have poor cardiometabolic outcome by the age of 5-6 years and by the age of 10 years.

**Methods:**

Data will be obtained from the Generation R (n=7893) and Prevention and Incidence of Asthma and Mite Allergy (PIAMA; n=3963) cohorts, two Dutch prenatally recruited cohorts. We will select candidate predictors that can be assessed during the first visit and/or during subsequent visits to the CHC center or pediatrician, including sex; parental age, education level, and BMI; smoking exposure; ethnicity; birth weight; gestational age; breastfeeding versus formula feeding; and growth data through the age of 6 years. We will design dynamic prediction models that can be updated with new information obtained during subsequent CHC visits, allowing each measurement to be added to the model. Performance of the model will be assessed in terms of discrimination and calibration. Finally, the model will be validated both internally and externally using the combined cohort data and then converted into a computer-assisted tool called *Pro***C**OR (Prediction Of Child CardiOmetabolic Risk).

**Results:**

This is an ongoing research project financed by the Dutch government. The first results are expected in 2016.

**Conclusions:**

This study may contribute to the national implementation of digitized tools for assessing the risk of overweight and related cardiometabolic outcome in young children, enabling targeted primary prevention, ultimately yielding relevant health gains and improved resource allocation.

## Introduction

The prevalence of childhood overweight and obesity is increasing worldwide, including the Netherlands [1,2]. Hypertension or prehypertension and low high-density lipoprotein cholesterol (HDL-C) values are related adverse cardiometabolic risk factors that are also highly prevalent in children. The prevalence of hypertension is approximately 3.5%, 9%, and 19% among normal weight, overweight, and obese children, respectively [3-5]. Moreover, 6.2% of normal weight children have low HDL-C levels [6]. Overweight, hypertension, and low HDL-C level in childhood generally persist into adulthood, but are usually asymptomatic. Importantly, the presence of these risk factors in childhood can be detrimental, increasing the lifetime risk of developing severe health complications such as coronary heart disease and type 2 diabetes [7-14].

Once an individual becomes overweight, this condition is usually difficult to reverse, even among children, as changing one’s lifestyle is difficult, and people often revert to their old habits resulting in even poorer health due to weight fluctuations [15]. Therefore, the European Society of Cardiology states that *primary* lifelong prevention of overweight and related poor cardiometabolic outcome deserves high priority beginning at birth [16]. Primary preventive interventions are particularly important in children aged less than 6 years, as changes in body mass index (BMI) below this age are more predictive of later overweight and poor cardiometabolic outcome than BMI changes after this age [17,18]. Moreover, parental lifestyle interventions when the child is young are associated with improved long-term outcome [19]. In addition, the American Academy of Pediatrics found that early intervention can yield better success in children with high cardiometabolic risk [20,21].

The EPODE (Ensemble Prévenons l’Obésité Des Enfants) program in northern France is a universal primary approach designed to prevent overweight in children by involving the family unit and society as a whole, for example, by changing the environment and influencing social norms. The merits of this approach have clearly been demonstrated [22]. Therefore, the program was successfully adopted, converted, and implemented in the Netherlands as the Youth on Healthy Weight (in Dutch: *Jongeren op Gezond Gewicht*, or JOGG) program [23]. Despite the introduction of this program, however, the prevalence of overweight remains relatively high, particularly among children from a low socioeconomic background [24]. Therefore, in addition to this universal approach, there is increasing interest in targeting primary prevention to children with the highest risk of becoming overweight and developing related poor cardiometabolic outcome. These risks may be estimated using prediction models or so-called “clinical prediction rules,” which use the best possible combination of risk factors to calculate the likelihood of a specific outcome.

Prototypes of such prediction models have been developed in the Netherlands within two Dutch cohort studies - the Prevention and Incidence of Asthma and Mite Allergy (PIAMA) birth cohort study and the Terneuzen cohort study - and in other countries, including India [25]. The two aforementioned Dutch prototypes are based on the presence of several predictors at birth, including paternal and maternal BMI [26], and on the trajectory of BMI standard deviation scores (BMI SDS) from 2 to 6 years of age [27]. Both Dutch prediction models have fair performance, indicating that targeted primary prevention of overweight and related poor cardiometabolic outcome is feasible with the help of evidence-based prediction models [26,27].

Here, we present the design of our study, which aims to develop dynamic prediction models to estimate the risk of young children (up to 6 years of age) developing overweight, hypertension or prehypertension, low HDL-C levels, and/or a high total cholesterol to HDL-C ratio by early adolescence. The resulting prediction models will be converted into easy-to-use tools called *Pro* **C**OR (PRediction Of Child CardiOmetabolic Risk), which can be applied both in the Dutch preventive Child Health Care (CHC) system and by pediatricians.

Because the Dutch CHC reaches more than 95% of all children of all ethnicities through 16 regular visits from birth until the age of 6 years, this approach will enable targeted primary prevention for children in this age group. The *Pro* **C**OR tools will provide risk estimations by taking into account the accumulation and changes in exposure to multiple risk factors throughout life, including familial risk factors [28], smoking exposure, physical inactivity, and changes in BMI [29].

Because we cannot exclude the possibility that children may already have an adverse cardiometabolic profile at this young age, thereby indicating the need for treatment, our secondary aim is to develop risk estimation models to assess the need for further diagnostics of poor cardiometabolic outcome at age 5-6 years and at age 10 years, two ages at which regular visits are made to the Dutch CHC centers.

The study design includes (1) data collection; (2) the development, external validation, and, if required, updating of dynamic prediction rules within two large Dutch cohort studies; and (3) an assessment of whether the resulting tools can be implemented in practice. Therefore, the study will also focus on risk communication, as this is an important aspect that can affect the likelihood of successful implementation.

The outcomes of the prediction rules will be assessed in early adolescence, because BMI SDS values measured between 10 and 18 years of age are highly correlated with adult BMI SDS values and body fat mass [17]. Moreover, as discussed above, hypertension/prehypertension, low HDL-C levels, and high total cholesterol levels often persist from childhood into adulthood, particularly from adolescence [10-12]. Our study proposal has been awarded a TOP Prevention Grant from ZonMw (title: “Targeted primary prevention of overweight and cardiometabolic risk using dynamic risk assessments from infancy onward in Child Health Care”; 200500006) , offering the opportunity to innovate in terms of both content and collaboration. See Multimedia Appendices 1, 2 and 3 for the reviewers’ comments on the original version of the research proposal.

## Methods

### Stakeholder Group

For the study results to be useful, the developed tools must ultimately be implemented in practice. Therefore, a group of stakeholders has been established and comprises CHC physicians and nurses, pediatricians, general practitioners, dieticians, epidemiologists, programmers, scientists, and policy makers. In several phases of the study, parents will also be involved to ensure maximum communication regarding risk assessments with parents and maximum implementation of the *Pro* **C**OR tools in the CHC.

### Design and Setting

This study is embedded in the Generation R (n=7893) and PIAMA (n=3963) cohorts. These large, longitudinal Dutch cohorts provide independent datasets with many uniform variables regarding fetal life, pregnancy, birth, and early growth.

The Generation R study is a population-based, multiethnic cohort study composed of children born to 9778 mothers living from 2002 through 2006 in Rotterdam, the Netherlands [30]. Of all children who were eligible at the time of birth (n=7893), 61% participated in the follow-up study. In this cohort, children of diverse ethnic origins are well represented, including Caucasian (56%), Surinamese (9%), Turkish (7%), and Moroccan (6%) children. Three questionnaires were sent to the participating mothers during their pregnancy. Questionnaires were also sent when the child was 2, 6, 12, and 18 months of age and 2, 3, 4, 5, and 7 years of age. During pregnancy, the participants visited the research center in early, mid, and late pregnancy. From birth through 5 years of age, data regarding growth, development, and medical conditions among the Generation R participants were collected during their routine visits to the CHC centers. At the age of 5-6 years, all participating children and their parents were invited to visit the research center for a medical examination. The data collection of children 9-10 years of age was recently completed.

The PIAMA birth cohort is a population-based prospective cohort comprising 4146 women who were pregnant at baseline [31]. Because 183 women were lost to follow-up before any data regarding the child were collected, the study began with 3963 newborn children who were born in 1996-1997 in various regions of the Netherlands. Questionnaires were sent to the participants during pregnancy, when the child was 3 months of age, annually thereafter from 1 through 8 years of age, and at 11, 14, and 17 years of age. At 8, 12, and 16 years of age, subgroups of participating children were invited for a medical examination. The cohort consists primarily of Caucasians and includes children from both urban and rural areas [31].

Both the Generation R study and the PIAMA study were conducted in accordance with the guidelines established by the Declaration of Helsinki and were approved by the respective medical ethics committees (MECs). Written informed consent was obtained from all participating parents. The MEC for the Generation R study was the MEC of Erasmus Medical Center (MEC 217.595/2002/202). The MECs of the participating institutes for the PIAMA cohort were MEC Rotterdam (132.636/1994/39, 137.326/1994/130 and P04.0071C/MEC 2004-152), MEC Groningen (94/08/92, P04.0071C/ M 4.019912, June 28, 2004, and 12-019/K), and MEC-TNO Utrecht (95/50, February 28, 1996, CCMO Utrecht P000777C, 2000, P04.0071C, 2004, 07-337/K, May 20, 2008, and 12-019/K, May 25, 2012).

### Overweight and Poor Cardiometabolic Outcome

The outcomes of the dynamic prediction models measured at age 10-15 years are as follows:

- overweight, defined using the International Obesity TaskForce (IOTF) cutoff values [32];
- abdominal overweight, defined based on the findings of the Fifth National Growth Study in the Netherlands, which will be available in the near future [2];
- hypertension or prehypertension, based on systolic and diastolic blood pressure and defined by the National High Blood Pressure Education Program Working Group on High Blood Pressure in Children and Adolescents [33]; and
- low HDL-C level or high total cholesterol to HDL-C ratio, in which low HDL-C is defined as <0.9 mmol/L and high total cholesterol is defined as >6.3 mmol/L. Because total cholesterol to HDL-C ratio in childhood quite accurately predicts subclinical adult atherosclerosis, including coronary heart disease, it will be useful to obtain both total cholesterol and HDL-C values [13,14].

The outcomes of the risk estimation models at ages 5-6 years and 10 years are hypertension or prehypertension and a low HDL-C level and high total cholesterol to HDL-C ratio, according to the aforementioned definitions.

### Candidate Predictors

The candidate predictors that were used to develop the two Dutch prototypes [26,27] will also be used to develop the new prediction models. In addition, other candidate predictors will be selected from the literature, including relatively novel risk factors such as child’s height and gross motor skills [34,35]. The stakeholder group will also provide advice—via discussion rounds—regarding the final selection of clinically relevant candidate predictors. The candidate predictors can be divided into the following two categories: (1) predictors that are currently routinely collected by the CHC and (2) predictors that may be routinely collected by the CHC in the near future.

The candidate predictors for the dynamic prediction models are as follows:

- Demographics and general characteristics

Sex and ethnicity of the child, urbanization grade, parity of the mother, and the education level and age of both parents.

- Prenatal/perinatal factors

- Child characteristics: birth weight, gestational age, head circumference, Apgar scores (at 1 and 5 minutes), meconium in the amniotic fluid, formula feeding immediately after birth.
- Parent characteristics: cesarean or vaginal delivery, use of vitamins and/or minerals by the mother, smoking (including second-hand smoke exposure), BMI of the mother before and after pregnancy, BMI of the father, and blood pressure of both parents.

- At the age of 3 months

Body weight and body length, smoking exposure, and breast milk and/or formula.

- At the age of 1 year

Body weight and height, smoking exposure, breast milk and/or formula, data from the food frequency questionnaire (FFQ), dietary salt, and BMI of the mother.

- At the age of 5-6 years

Body height and weight, smoking exposure, FFQ, dietary salt, and physical activity.

- Between birth and the age of 6 years

- Periodic measurements of height and weight, collected by the CHC.
- Periodic measurements of gross motor skills, collected by the CHC.

For the prediction models to estimate the risk of poor cardiometabolic outcome, with the aim to assess the need for further diagnostics (blood pressure measurements and blood tests), the aforementioned candidate predictors will be used at the age of 5-6 years. At 10 years of age, the growth measurements and any other variables that can vary over time such as passive smoking between 6 and 10 years of age will be included as well.

### Development of the Prediction Models

Dynamic risk prediction models will be developed in both cohorts. Missing values will be taken into account using advanced imputation techniques [36].

To design the dynamic risk prediction models, we will choose a statistical technique that can account for clustering of the data due to the collection of repeated measurements over time within children. This approach makes it possible to introduce repeatedly assessed risk factor information for children between birth and the age of 6 years into the model. For comparison purposes, the initial model (created at birth) will be updated with new information obtained at each visit, including the age of the child [37].

The statistical technique used in this study will be determined based on a comparison between existing simple and advanced statistical methods in order to handle repeatedly measured independent variables and a non-time-varying outcome variable in a prediction model, as described by Chen et al [38] and Tu et al [39]. We will compare the applicability of these methods for the development of prediction models for use by epidemiologists and clinical practitioners, and we will test the predictive quality of these models.

Backward selection will determine the final risk prediction models. The initial regression model, including all potential predictors, will be fitted in the dataset. To ensure sufficient power during the modeling process, we will optimize the balance between the number of variables and outcome “events” in the models [40].

Next, variables that have weak associations will be omitted from the model. We will use the Akaike information criterion (AIC) as a stopping rule to determine which variables should be removed from the model. For a single predictor, AIC equates to selection at *P*=.157 [40]. This relatively high *P* value results in the inclusion of weaker predictors in the model at the cost of potentially selecting a nuisance variable. Such a model usually performs well in new subjects [40].

### Performance of the Prediction Models

The performance of the prediction models will be studied in terms of both discrimination and calibration. Discriminative ability expresses how well the model can distinguish between preadolescent children with the outcome and preadolescent children without the outcome. Discriminative ability will be assessed with the area under the receiver operating characteristic curve [41]. To reflect how well the predicted and observed values agree, the calibration slope will be estimated in a regression model using the linear predictor as the sole variable. The linear predictor is calculated by multiplying the model’s regression coefficients with the predictor values for each child and then summing these values (including the intercept). Under ideal conditions, this slope is 1, which means that the predicted and observed probabilities agree over the entire range of predictions [42]. Furthermore, the explained variability will be assessed, providing an overall measure of performance [43]. We will also decide on the cutoff values to be used in practice, taking into account sensitivity, specificity, and positive and negative predictive values for different categories of predicted probabilities.

### Validation and Updating

We will use bootstrapping techniques to internally explore the need to correct for optimism in regression coefficients and performance measures of the dynamic risk prediction models. The dynamic risk prediction models, which will be developed in the oldest cohort (PIAMA), will be externally validated in the youngest cohort (Generation R). Validation is essential in order to determine the ability of a model to reliably predict the outcome in other populations and settings. If necessary, the models will be updated based on the validation results in both cohorts. Thus, we will study whether the models either remain stable or improve by removing or adding predictors that are available in the validation data. Finally, the model will be fit to the combined PIAMA and Generation R data, with the goal of achieving optimal precision of the estimated coefficients.

### Development and Pilot Testing of the Prediction Tools

After the models have been developed, they will be converted into computer-assisted tools (the *Pro* **C**OR tools). These tools will be easily applicable in CHC, providing for repeated risk assessments, thereby aiding CHC professionals in their decision to give advice, offer extra follow-up consults, and/or refer children and their parents for preventive intervention or further diagnostics. By consulting with experts in a Delphi study, we will determine which cutoff values will be used for these actions, with the aim of achieving the optimal balance between sensitivity, specificity, positive and negative predictive values, and the perceived diagnostic and therapeutic burden for the parents and their children. Finally, the feasibility of the implementation will be assessed in a pilot study to be conducted in three CHC centers within the Netherlands. In this pilot, we will study the reach, frequency, and percentage of children with a predicted high risk of overweight and/or poor cardiometabolic outcome, acceptability of risk communication and risk assessments, satisfaction, the potential for stigmatization, protocol compliance, advice and referrals, maintenance, and time spent. For this purpose, questionnaires will be developed both for parents and for CHC professionals. Approval will be requested from the MEC before the start of the pilot study.

## Results

This is an ongoing research project, and the first results are expected in 2016. The study began in December 2013 and is funded by the Health Research and Development Council of the Netherlands (ZonMw Grant no. 200500006).

## Discussion

Our study will provide an individualized approach for primary prevention of overweight and poor cardiometabolic outcome by combining risk estimates with the existing general approach. This individualized, targeted approach will bridge an existing gap in the primary prevention of overweight and related adverse cardiometabolic outcomes. This is likely both important and relevant, given that the effects of secondary prevention of overweight and obesity have been disappointing—even among younger children—particularly over the long run [44]. Moreover, the success of universal primary prevention has been less pronounced in groups from lower socioeconomic backgrounds [22,24]. Importantly, the *Pro* **C**OR tools will also enable the identification of children who already have high blood pressure and an abnormal lipid profile, thereby offering these children additional diagnostics and treatment options by their pediatrician; this is particularly relevant, as these adverse cardiometabolic outcomes tend to persist into adolescence and even adulthood, and are usually asymptomatic [10-12].

The success of the *Pro* **C**OR tools when applied in early childhood will be reflected in the possibility to refer children with increased risk to a primary preventive program. Although such primary preventive interventions are relatively scarce, and although the majority of efforts have focused on developing secondary preventive interventions, several studies have demonstrated the potential benefit of applying primary preventive interventions in this extremely young age group [45-47]. In addition, primary preventive interventions aimed at the most susceptible individuals are often more effective than interventions that target the entire population [48]. Therefore, participating JOGG cities in the Netherlands have opted to implement interventions that are similar to Mini-MEND, a program aimed at children and families with increased risk at the age of 2-4 (or 5) years; this program is currently being evaluated in Australia and the United States [49-51]. Mini-MEND is the younger-age equivalent of MEND (Mind, Exercise, Nutrition, Do It!), a family-based intervention that was designed for older children and was found to be cost-effective in a randomized controlled trial [52].

The *Pro* **C**OR tools can also be linked to Web-based content, including educational materials, advice, and/or referrals to evidence-based intervention programs, thus stimulating a healthy lifestyle and/or health care–seeking behavior. Finally, the risk estimates generated by the *Pro* **C**OR tools can also be used to study the effectiveness of interventions, as a risk assessment of future overweight and related cardiometabolic outcomes is likely to be more predictive than the currently used definition of overweight using the cutoffs established by the IOTF [53].

Other methods have been developed to identify children who are at high risk for becoming overweight and/or developing adverse cardiometabolic outcome; such methods include the use of a single risk factor, combining risk factors, and developing risk models, risk algorithms, and risk charts. For example, in both Sweden and the United States, risk charts have been developed to predict adult overweight based on a single BMI measurement during childhood [54,55]. Another study performed in the United States reported crude risk estimates of being overweight by the age of 12 years based on one, two, or three BMI values measured between 2 and 4½ years of age [56]. In the United Kingdom, an overweight risk algorithm was developed to identify at-risk children at age 3 years based on predictors that were assessed in the first year of life [57]. To date, however, most risk models have not been dynamic and barely took into account individual BMI development. Moreover, extremely high-performance prediction models are required before acceptable large-scale implementation is possible in practice.

A strength of our study is that we will include additional candidate predictors, thereby helping optimize the performance—and hence the cost-effectiveness—of these models. Moreover, performing repeat risk assessments enables clinicians to both adapt to and individualize the strategies for each child based on new findings. This approach may contribute to the realization of more tailor-made primary prevention programs, with the aim of reversing the high risk of overweight and adverse cardiometabolic outcome in preadolescence.

One potential limitation of our study is that the outcomes of the prediction models were examined in early adolescence; moreover, the cohort data do not currently offer the opportunity to study these outcomes at adulthood. However, the timing of puberty is associated with adult obesity and poor cardiometabolic outcome [58]. On the other hand, the correlation between BMI SDS at age 10 years and BMI SDS at young adulthood is high (>0.7), suggesting that preventing high BMI SDS by the age of 10 years is relevant in terms of health outcome at adulthood [17]. In future studies, we will expand the prediction models with health outcomes at later ages.

By providing longitudinal guidance to children, the Dutch CHC is highly specialized with respect to the growth and development of children, suggesting that performing dynamic risk assessments is a very feasible approach in this setting. Therefore, the Dutch CHC is ideally positioned to help improve resource allocation by identifying high-risk children and excluding children with relatively low risk.

Finally, the tools that emerge from this study will be made available via the Internet, provided that stakeholders—including parents and/or their representatives—voice the need for Web-based access to the tools.

A potential disadvantage associated with applying the *Pro* **C**OR tools is that it may cause harm due to stigmatization and parental and/or individual concerns. Therefore, we will investigate the effects of risk communication on possible unwanted side effects in the pilot study. At the same time, we must bear in mind that any potential harm due to the application of current cutoff points for overweight and/or obesity are currently unknown; moreover, the effectiveness of interventions that are based on risk assessments can only be studied after the prediction tools have been developed. Therefore, assuming that prediction tools with high sensitivity, specificity, and negative and positive predictive values can be developed, the next logical step would be to perform a randomized controlled trial designed to evaluate the effects of the prediction tools in combination with primary preventive intervention programs.

## Appendix

Multimedia Appendix 1 Review 1.

Multimedia Appendix 2 Review 2.

Multimedia Appendix 3 Review 3.

## Abbreviations

AIC: Akaike information criterion
BMI: body mass index
BMI SDS: BMI standard deviation scores
CHC: Child Health Care
FFQ: food frequency questionnaire
HDL-C: high-density lipoprotein cholesterol
IOTF: International Obesity TaskForce
JOGG: Jongeren op Gezond Gewicht (in English: Youth on Healthy Weight)
MEC: medical ethics committee
MEND: Mind, Exercise, Nutrition, Do It!
PIAMA: Prevention and Incidence of Asthma and Mite Allergy
ProCOR: PRediction Of Child CardiOmetabolic Risk
