# Supplementary material for: Prediction of Preadolescent Overweight and Poor Cardiometabolic Outcome in Children up to 6 Years of Age: Research Protocol
Source: JMIR Res Protoc. 2016 Jun 23;5(2):e85. doi: 10.2196/resprot.5158 (PMC4937175; doi:10.2196/resprot.5158)
Supplement: Multimedia Appendix 1 [file resprot_v5i2e85_app1.pdf]

Subsidieprogramma / Subsidy programme	:	**TOP subsidies**
Dossiernummer / Dossier number	:	**40-00812-98-12124**
Aanvrager / applicant	:	**Dr. M.L.A. de Kroon MD**
Projecttitel / Project title	:	**Targeted primary prevention of overweight and cardiometabolic risk using dynamic risk assessments from infancy onward in Child Health Care**
Beoordelingscode / Assessment code	:	**B.2012.01623**

1. TOP Grant application quality assessment form

Legenda: A+ (Highest quality), A (High quality), B (Good quality), UF (Unsuccessful in this form), U (Unsuccessfull)

1.1 Under 'elaborate' please give written comments on the following 5 criteria:

A+	A	B	UF	U
	X			

1. problem definition, objective, innovative potency
2. strategy
3. project group
4. feasibility
5. knowledge utilisation

*Always provide stronger and weaker points for **EACH** of these criteria (a SWOT analysis). Please check the accompanying 'guidelines' for a description of the criteria. You can use a free text format (no space limit) as long as it is clear what criterion you are discussing.*

Essential is an in depth explanation accompanying your critiques, thus enticing a written debate with the applicants.

Superfluous evaluations do not provoke applicants to defend or elaborate on their ideas and plans (through rebuttal) and are of little use to the committee.

Fundable or not fundable? Please complete your assessment by choosing one of the following five grades:

(check the accompanying 'guidelines' for further explanation)

A+, Highest quality, significance and recommendation for funding
Funding is highly recommended

A, High quality, significance and recommendation for funding
Funding is recommended

B, Good quality and significant
Funding is recommended only if ample resources are available

UF, Unsuccessful in this form
Funding of the proposal in its present form is not recommended

U, Unsuccessful
Funding is not recommended

Please justify your final assessment by summarizing or briefly commenting on the strengths and weaknesses of the proposal.

1. problem definition, objective, innovative potency

Cardio-metabolic disease remains the #1 medical problem facing the world. It has been well documented that the processes leading to cardiovascular disease and type 2 diabetes begins early in life. Identifying youth that may benefit most from primary (and primordial) prevention of risk factors that contribute to the development of such diseases is paramount if the burden of these diseases is to be reduced. But how do we realize this issue in practical terms and how do we implement a system of identification and treatment? The US NHLBI recently announced comprehensive guidelines for cardiovascular health and risk reduction in the pediatric setting. This is an ambitious project that has already been criticized either for some of the recommendations or indeed, the (in)ability to implement it. This proposal by de Kroon and colleagues is an extremely timely proposal for a much needed tool in the clinical setting. The study has the potential to change clinical practice and provide an avenue for

directing key prevention efforts (to individuals at highest risk). What is proposed is inherently more simplified than those of the NHLBI and by this, I would expect the translation from idea through to implementation (as the investigators outline) to be highly likely.

2. Strategy
I could not identify any major weaknesses for the design, methods, or analyses proposed. I did have some concerns around conceptual weaknesses of the proposal that I thought were not given due consideration in the proposal.

-While the importance of BMI, blood pressure and lipids are demonstrated, the reality is, the entire concept of this proposal is based on prediction (and implementation of preventive systems) of a risk factor that is a predictor of later cardio-metabolic diseases. Clearly, the outcomes proposed are not hard end-points and the investigators should at least acknowledge this and argue why the risk factors they propose to identify are robust replacements for, say, evidence of hard clinical outcomes. In this, the authors should note perhaps the need for lengthy follow-up to collect sufficient hard end-points and why that may not be feasible given current knowledge on the topic. The authors may also wish to discuss the potential for modifying (refining) their tool as hard end-points become available within their cohorts.

-The investigators did not consider data from other prospective cohorts (Bogalusa Heart, Young Finns) and an international consortium (e.g. Juonala & Magnussen et al. Circulation 2010) that tends to suggest that the association between early presentation of risk factors and later cardio-metabolic outcomes in adulthood is much stronger for adolescents (older children) compared with (younger) children. Identifying such risk factors at early age, as the investigators propose, may prove less useful (utility) than if measures where collected in children of older age. The investigators may argue based on previous evidence for tracking. But even the best tracking risk factor, overweight/obesity, only sees roughly ½-3/4 track into adulthood. While this is a significant amount, one could also say that ~1/4 (best case) do not track, and given the wide-reaching population of this proposal, this number is not insignificant. HDL-C levels also appear to track well, but blood pressure levels have been shown to track substantially less well than lipids and adiposity.

-In section B.1, the PIAMA cohort is to be re-contacted. There will be non-respondents. Can the investigators provide an indication as to what proportion of the PIAMA cohort they expect to get consent from?

-It was not clear if the risk tool will provide separate predictions for all risk factors (i.e. overweight, pre-hypertension, low HDL-C) or a single prediction of future ‘at-risk’ status based on presence of any one of these risk factors. I assume the former if individualized intervention/prevention is to be realized. Also, why are other potential outcomes not considered? LDL-C/non-HDL-C for example?

-In section B.2.3, a minor issue, the NHBPEP define pre-hypertension as >=90th percentile for gender, age and height, not the “85th” as stated.

-Have the authors considered gene data in the derivation of the risk tool? Current indications using SNPs or gene risk scores provide little evidence of major gains in predictiveness when such are taken into account, even when child-adult prediction of adult cardio-metabolic outcomes is considered. This would also severely limit the ‘simplicity’ of any clinical tool. Though I support gene ‘non-inclusion’ the authors may wish to comment on this further.

3. project group
It is clear that this group is extremely capable of carrying out the work they proposal. Short of an international consortium, one would be hard-pressed to find a better representation of key individuals in a single country with demonstrable links to the area. The group has demonstrated long-term links with the study’s in question, and analysis and write-up of data on the broader topic of risk prediction/association using longitudinal data collected in childhood spanning to adulthood. The statistical strength of the group is high and again demonstrable within the area of this proposal.

4. Feasibility
The investigators have proposed a workable design, with key people leading the study, and most importantly have demonstrated collaborative arrangements with relevant stakeholders. Because of this I have no reservations concerning the proposed work and the timeline for implementation.

5. knowledge utilization
Perhaps the primary strength of this proposal is the linkage with key partners that will help realize the potential of the tool/system this proposal first aims to develop, then implement, then assess. In research and clinical terms, it is

very obvious the proposal has strong merit – the difficulty is in taking good research to the next, translational stage - to the clinic setting. The investigators, as they have outlined, have strong links that will help realize the true potential of this project – ultimately, implementation in a clinical setting and the changing of clinical practice.
