# Supplementary material for: Prediction of Preadolescent Overweight and Poor Cardiometabolic Outcome in Children up to 6 Years of Age: Research Protocol
Source: JMIR Res Protoc. 2016 Jun 23;5(2):e85. doi: 10.2196/resprot.5158 (PMC4937175; doi:10.2196/resprot.5158)
Supplement: Multimedia Appendix 2 [file resprot_v5i2e85_app2.pdf]

Subsidieprogramma / Subsidy programme	:	**TOP subsidies**
Dossiernummer / Dossier number	:	**40-00812-98-12124**
Aanvrager / applicant	:	**Dr. M.L.A. de Kroon MD**
Projecttitel / Project title	:	**Targeted primary prevention of overweight and cardiometabolic risk using dynamic risk assessments from infancy onward in Child Health Care**
Beoordelingscode / Assessment code	:	**B.2012.01442**

1. TOP Grant application quality assessment form

Legenda: A+ (Highest quality), A (High quality), B (Good quality), UF (Unsuccessful in this form), U (Unsuccessfull)

1.1 Under 'elaborate' please give written comments on the following 5 criteria:

A+	A	B	UF	U
		X		

1. problem definition, objective, innovative potency
2. strategy
3. project group
4. feasibility
5. knowledge utilisation

*Always provide stronger and weaker points for **EACH** of these criteria (a SWOT analysis). Please check the accompanying 'guidelines' for a description of the criteria. You can use a free text format (no space limit) as long as it is clear what criterion you are discussing.*

Essential is an in depth explanation accompanying your critiques, thus enticing a written debate with the applicants.

Superfluous evaluations do not provoke applicants to defend or elaborate on their ideas and plans (through rebuttal) and are of little use to the committee.

Fundable or not fundable? Please complete your assessment by choosing one of the following five grades:

(check the accompanying 'guidelines' for further explanation)

A+, Highest quality, significance and recommendation for funding
Funding is highly recommended

A, High quality, significance and recommendation for funding
Funding is recommended

B, Good quality and significant
Funding is recommended only if ample resources are available

UF, Unsuccessful in this form
Funding of the proposal in its present form is not recommended

U, Unsuccessful
Funding is not recommended

Please justify your final assessment by summarizing or briefly commenting on the strengths and weaknesses of the proposal.

1. Problem definition, objective, innovative potency

Strengths
i) The applicants present an interesting concept which uses already collected national data to determine if adolescent overweight can be predicted with more accuracy when children are only 0 to 6 years of age. The basis of the idea is sound and important - it seems that general obesity prevention initiatives are not enough for those who are already overweight - more targeted intervention is required.

Weaknesses
i) I felt the application would have been significantly improved by providing more detail as regards the strength of

the exisiting date. eg. general terms such as "moderately correlated", "track" etc do not give the reader an idea of exactly HOW strong these relationships are. If current tracking is 0.2 for instance, is this useful? What if it is 0.6 - more likely therefore that intervention could benefit.
ii) I was not convinced that aim 2 was particularly novel nor innovative. US pediatric recommendations have now suggested for some time that screening initiatives should also address metabolic health - how does this application really differ?
iii) Additional detail on the existing models would have allowed a better evaluation of the proposed models - these seemed in part a bit of a fishing expedition using data that existed - rather than purposively collecting hypothesised data. Why use maternal height if maternal BMI is available? Surely the latter is more useful than the former? How well do gross motor skills track and has this been associated with weight status?

2. Strategy
Strengths
1. Multifaceted approach suggested including literature review, examination of current protocols, development and validation in two cohorts and piloting of "new" protocol and decision tree.
2. In general the methods appeared appropriate and comprehensive, with the exception of the few queries outlined below.

Weaknesses.
1. I wasn't clear why backward elimination using GEE is being used - not typical?
2. More detail on the "short" questionnaire used in part D would have been useful. The authors should justify why more in depth qualitative work to ascertain reach and compliance was not chosen - it would seem to me that they are trying to assess quite complicated issues from a "brief" questionnaire.
3. Does it matter how many measurements you have per child? It is difficult to imagine how the clinical tool is used practically and how you weight the information obtained at different time points, so that each time, a child has a new risk score calculated.
4. If the two cohorts produce very different cross-validations (which is a reasonable expectation) how do you "update" the algorithm - specific example might have been useful.

3. Project group.
Strengths
1. Very experienced PI with extensive publication record - rest of the team appears more junior (relatively speaking) but still with impressive papers and publication records - many in high impact journals.
2. Has experience with the general area - PI very experienced in longitudinal datasets which is important given the more complex analyses involved.

Weaknesses
1. Not clear how will do the complicated statistical analyses? Assume it is Dr Heymans?

4. Feasibility
Strengths
1. Fact that the data have essentially already been collected - or will be soon
2. Working with the existing child care framework
3. Inclusion of a very comprehensive stakeholder group - although in practice such a large group may pose difficulties.
4. Facilities apppear adequate and in place.
5. The project timeline appears reasonable - although I am not entirely sure that there is enough time for the final piloting phase - this could be quite comprehensive

5. Knowledge utilisation
Strengths
1. Have listed scientific papers, student theses, dissemination through teaching to medical students, presentation at meetings.

Weaknesses
1. Some indication of what the 12 expected publications might be would be useful - how did the applicants arise at this number? What aspects of the project are particularly novel and therefore more likely to be published?
