# Supplementary material for: Prediction of Preadolescent Overweight and Poor Cardiometabolic Outcome in Children up to 6 Years of Age: Research Protocol
Source: JMIR Res Protoc. 2016 Jun 23;5(2):e85. doi: 10.2196/resprot.5158 (PMC4937175; doi:10.2196/resprot.5158)
Supplement: Multimedia Appendix 3 [file resprot_v5i2e85_app3.pdf]

Subsidieprogramma / Subsidy programme	:	**TOP subsidies**
Dossiernummer / Dossier number	:	**40-00812-98-12124**
Aanvrager / applicant	:	**Dr. M.L.A. de Kroon MD**
Projecttitel / Project title	:	**Targeted primary prevention of overweight and cardiometabolic risk using dynamic risk assessments from infancy onward in Child Health Care**
Beoordelingscode / Assessment code	:	**B.2012.0148B**

1. TOP Grant application quality assessment form

Legenda: A+ (Highest quality), A (High quality), B (Good quality), UF (Unsuccessful in this form), U (Unsuccessfull)

1.1 Under 'elaborate' please give written comments on the following 5 criteria:

A+	A	B	UF	U
		X		

1. problem definition, objective, innovative potency
2. strategy
3. project group
4. feasibility
5. knowledge utilisation

*Always provide stronger and weaker points for **EACH** of these criteria (a SWOT analysis). Please check the accompanying 'guidelines' for a description of the criteria. You can use a free text format (no space limit) as long as it is clear what criterion you are discussing.*

Essential is an in depth explanation accompanying your critiques, thus enticing a written debate with the applicants.

Superfluous evaluations do not provoke applicants to defend or elaborate on their ideas and plans (through rebuttal) and are of little use to the committee.

Fundable or not fundable? Please complete your assessment by choosing one of the following five grades:

(check the accompanying 'guidelines' for further explanation)

A+, Highest quality, significance and recommendation for funding
Funding is highly recommended

A, High quality, significance and recommendation for funding
Funding is recommended

B, Good quality and significant
Funding is recommended only if ample resources are available

UF, Unsuccessful in this form
Funding of the proposal in its present form is not recommended

U, Unsuccessful
Funding is not recommended

Please justify your final assessment by summarizing or briefly commenting on the strengths and weaknesses of the proposal.

Problem definition, objective, innovative potency and strategy:
Obesity in children, adolescents as well in adults has become a great health problem in westernized countries. In general the trends have been increasing, although in U.S. the latest reports tell, that the prevalences are leveling off. This study plan is addressed to an important problem.
In this study proposal the main target is to develop a better working screening program for children using the experience gained from the Terneuzen Birth Cohort.
The main idea is to find out those children, who will develep to be obese adolescents and/or who have cardiometabolic risk factors and when needed give them advice, offer extra follow-up consults or guide them to further diagnostics or offer them primary (and possibly also) secondary prevention.
The final screening program will be developed from the Terneuzen Birth Cohort, the PIAMA birth cohort and The

Generation R study.
The most difficult problem in obesity is the treatment.
One can ask, how much better results we can achieve by improving "diagnostics" or screening of subjects at risk, if we do not know, how to proceed after finding the risk subjects.
One critical point is, whether we should use screening and treat those, who are found, or should we use more general approach.
The STRIP study with a RCT design(see e.g. Lapinleimu H et al, Lancet 1995), which was mainly started for CHD-prevention has also aimed to prevent childhood and adolescent obesity and has succeeded in principle (Hakanen M et al Int J Obes 2006), although the results are far from ideal.
Especially the efforts against morbid obesity (this is most clearly "secondary prevention of obesity") are very demanding and the long-term results are often very disappointing, because permanent change of life-habits is so difficult.
Are the applicants sure that a common model can be made for these two cohorts, PIAMA children born in 1996-1997 and the Generation R children born in 2002-2006 ?
The Terneuzen Study suggests that the critical time is between ages 2 to 6 years.
In the PIAMA study there was no evidence for a specific
critical period for the development of overweight between birth and the age of 8 years, which challenges somewhat the conclusions from the Terneuzen study.
I doubt that secular trends in prevalence of overweight (= more obese children in Generation R than in PIAMA) may cause some problems, although perhaps the biggest change in obesity prevalences may have already been passed.
I would have liked to see also innovative approaches to the treatment of childhood obesity already at this stage of the project, because it is difficult to judge the benefit without seeing any changes of prevalences of overweight and obesity.
The results of this project should be compared e.g. with general approch applied in the STRIP-project to find out, whether screening and treating (this project) is superior to general approch with individualized advicing by the same persons (the STRIP design).
One of the main strengths of this application is that the researchers have access to digitalized information of growth data.
It is true that obesity (weight and height) tracks well.
Height and weight track far better than blood pressure (well-known already from e.g. Muscatine Study in 1970's). It is also known that serum cholesterol and HDL-cholesterol, track reasonably well (much better than blood pressure), but puberty modifies greatly HDL-cholesterol level, especially in boys.
Therefore I have strong doubts of using the fixed level of <0.9 mmol/l for HDL-cholesterol in both boys and girls aged 10-15 years of age. It would be more fair to use percentile values for age and gender (and possibly pubertal stage) in children aged 10-15 years

The project group:
The Project group has wide experience in growth studies including intra-uterine growth, growth in childhood and adolescence, physical activity, development of overweight and obesity and cardiovascular risk factors,
Also they have very good experience in biostistics and longitudinal data analysis (especially professor Twisk)
Dr. de Kroon (coordinator of the project) has gained experience from the Terneuzen Study, but I was a little surprised that in Web of knowledge (Thomson Reuters) the articles of Terneuzen study are not more widely cited.

Feasibility:
I have no serious doubts about feasibility of the project.
The Data is there, much work is needed. The main part of the budget is spent for 2 "promovendus" and one "programmeur", which are of couse essential for succesfull performance of the study.

Knowledge utilisation:
The results can and will be utilized in Dutch Health Care.
Definitively, this approach has good possibilities to produce at least a better prediction model.
I am just a bit worried, whether this "better case finding" will finally result in smaller prevalence of obese children, because better strategies to intervene and treat childhood overweigth and obesity are badly needed worldwide.
